# Increased heterogeneity of brain perfusion is an early marker of central nervous system involvement in antiphospholipid antibody carriers

**DOI:** 10.1371/journal.pone.0182344

**Published:** 2017-08-01

**Authors:** Ting-Syuan Lin, Pei-Ying Hsu, Chin-Hao Chang, Chi-Lun Ko, Yu-Min Kuo, Yen-Wen Wu, Ruoh-Fang Yen, Cheng-Han Wu, Ko-Jen Li, Yenh-Chen Hsein, Song-Chou Hsieh

**Affiliations:** 1 Department of Internal Medicine, National Taiwan University Hospital, Yun-Lin Branch, Yun-Lin, Taiwan; 2 Institute of Clinical Medicine, National Taiwan University College of Medicine, Taipei City, Taiwan; 3 Department of Nuclear Medicine, National Taiwan University Hospital, Yun-Lin Branch, Yun-Lin, Taiwan; 4 Department of Nuclear Medicine, National Taiwan University Hospital and National Taiwan University College of Medicine, Taipei City, Taiwan; 5 Department of Medical Research, National Taiwan University Hospital, Taipei City, Taiwan; 6 Department of Internal Medicine, National Taiwan University Hospital, Taipei City, Taiwan; 7 Cardiology Division of Cardiovascular Medical Center and Department of Nuclear Medicine, Far Eastern Memorial Hospital, New Taipei City, Taiwan; 8 Yang-Ming University School of Medicine, Taipei City, Taiwan; 9 Department of Laboratory Medicine, National Taiwan University Hospital, Yun-Lin Branch, Yun-Lin, Taiwan; Universite de Bretagne Occidentale, FRANCE

## Abstract

**Objective:**

The non-criteria neuropsychiatric manifestations of antiphospholipid syndrome include headache, dizziness, vertigo, seizure, depression and psychosis. There were still no objective methods qualified to detect the early central nervous system involvement in non-criteria antiphospholipid syndrome. We evaluated the effectiveness of Tc-99m ECD SPECT in assessing circulatory insufficiency in the brains of patients with antiphospholipid antibodies and neuropsychiatric symptoms but without thromboembolism.

**Materials and methods:**

Patients with a history of positive antiphospholipid antibodies and neuropsychiatric symptoms composed the case group; patients without antiphospholipid antibody served as the control group. Subjects with a history of thromboembolism or autoantibodies to extractable nuclear antigens were excluded. All patients received Tc-99m ECD SPECT studies and were classified by the number of positive antiphospholipid antibodies they carried. The heterogeneity of brain perfusion was defined as the coefficient of variation of the SPECT signals. Analysis of variance (ANOVA) was applied to evaluate the differences between the groups.

**Results:**

Total 60 adult patients were included in this study. There were 54 patients in the case group and 6 patients in the control group. The mean age was 38.3 ± 11.5 years. There were 52 women and 8 men. There was no significant difference in the mean brain perfusion between groups (*P* = 0.69). However, Tc-99m ECD SPECT demonstrated significant heterogeneity of brain perfusion in relation to the number of antiphospholipid antibodies (*P* = 0.01).

**Conclusions:**

This is the first study demonstrating that Tc-99m ECD SPECT can early detect the increased heterogeneity of brain circulation in non-criteria antiphospholipid antibody carriers.

## Introduction

Antiphospholipid syndrome (APS) is characterized by recurrent thromboembolism and miscarriages among young adults [1, 2]. Classification of APS requires evidence of clinical events (vascular thrombosis and/or adverse obstetric events) and repeated presence of antiphospholipid antibodies [3]. Additionally, the non-criteria presentations of APS, such as nephropathy, valvulopathy and neuropsychiatric symptoms can also adversely affect patients’ quality of life and work capacity [4]. The neuropsychiatric presentations of APS include headache, dizziness, vertigo, seizure, depression and psychosis; affected individuals could exhibit these symptoms before they develop thromboembolism [5–8].

Brain magnetic resonance imaging(MRI) can identify the lesions of cerebrovascular accidents but often yields negative results in non-criteria APS patients [9]. Previous studies reported that single photon emission computed tomography (SPECT) could detect the decreased brain blood flow in APS patients with a history of thrombotic events [10, 11]. Nonetheless, the usefulness of brain SPECT for assessing non-criteria APS is unclear. Therefore, we evaluated whether Tc-99m ethyl cysteinate dimer (ECD) SPECT is useful for assessing circulation insufficiency in the brains of patients with antiphospholipid antibodies and neuropsychiatric symptoms but without thromboembolism.

## Materials and methods

### Patients

This is a retrospective study based on a review of the electronic records of patients received Tc-99m ECD brain SPECT during 1st November 2004 to 30th June 2016. The case group comprised adults (age ≥18 years) with a history of positive antiphospholipid antibodies and neuropsychiatric symptoms; patients with neuropsychiatric symptoms but without antiphospholipid antibodies served as the control group. Patients were excluded if they had a history of thromboembolism, which was defined as cerebrovascular accidents, myocardial infarction, unstable angina, peripheral arterial occlusion, deep venous thrombosis and pulmonary embolism. Subjects were also excluded if they had positive antibodies to extractable nuclear antigens, including double stranded DNA, Ro/SSA, La/SSB, Sm, ribosomal P, Scl-70, centromere, Jo-1, RNP, RNA polymerase III, PM/Scl, fibrillarin, PCNA and Mi-2. The collected parameters included age, gender, weight, height, status of smoking, clinical presentations and laboratory tests. The brain computed tomography and magnetic resonance imaging were reviewed. The patients were grouped by the number of positive antiphospholipid antibodies they carried. This study was approved by Research Ethics Committees and Institutional Review Board of National Taiwan University Hospital (NTUH REC Number: 201409053 RINA). The institutional review board did not mandate patient consent, since patient records was de-identified prior to analysis, and patient privacy was not breached.

### Antiphospholipid antibodies

The antiphospholipid antibodies measured in this study included IgG/IgM to cardiolipin, β2-Glycoprotein I (β2GPI), phosphatidylserine and phosphatidic acid; these were tested by ELISA procedures. The commercial kits which included QUANTA Lite ACA IgM III, EliA Cardiolipin IgG, EliA β2-Glycoprotein I IgG and APhL ELISA IgG/IgM HRP Kit were used. The ACA antigens were cardiolipin and bovine β2GPI; the cut-off values were 12.5 MPL for ACA IgM and 10 GPL for ACA IgG. The antigen of anti-β2GPI IgG was human β2GPI; the cut-off value was 10 U/ml. The antigens of APhL were phosphatidylserine, phosphatidic acid and β2GPI; the cut-off values were 15 MPL for APhL IgM and 15 GPL for APhL IgG. Lupus anticoagulants (LA) were detected by the simplified Dilute Russell’s Viper Venom Test; LA was considered positive if the ratio of the LA1 screen test to LA2 confirmation test was greater than 1.5. All procedures were performed in strict adherence to the manufacturers’ instructions.

### Tc-99m ECD brain SPECT

**Fig 1:**
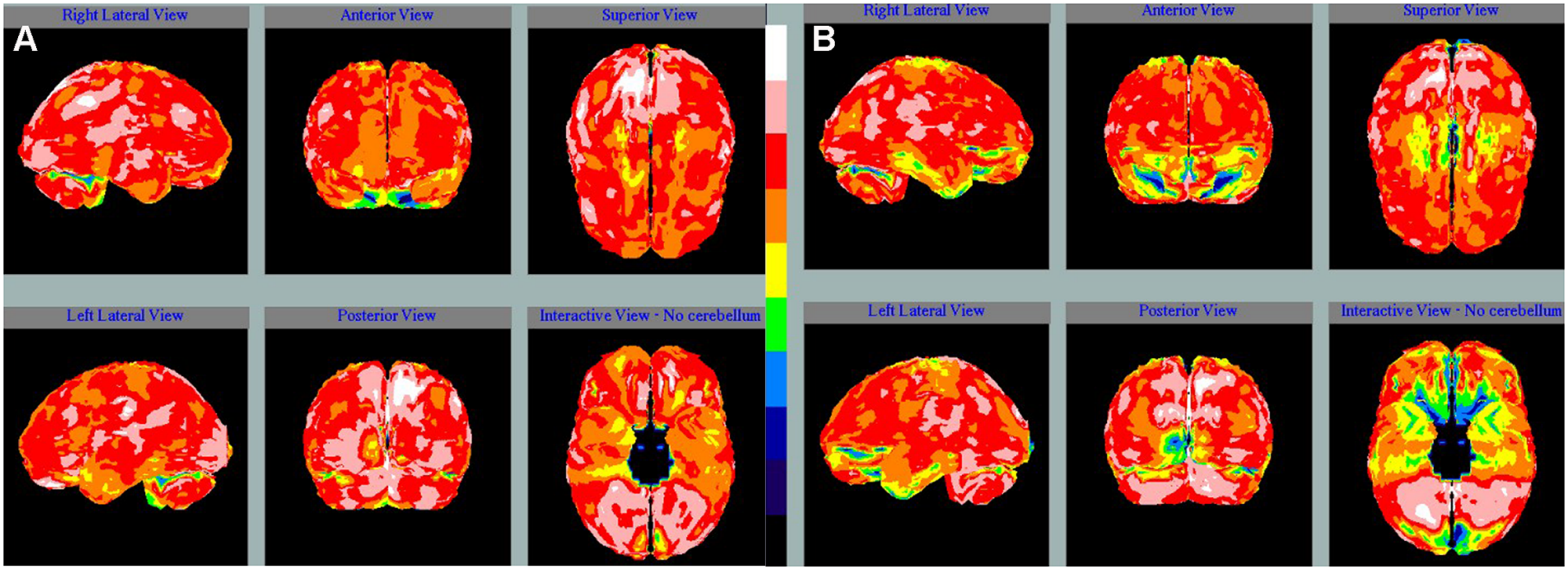
The images of Tc-99m ECD brain SPECT of two patients. (A) A 50-year-old female had occasional dizziness for one year. Brain MRI did not reveal any definite organic brain change. The autoimmune screen showed no positive autoantibody including antiphospholipid antibody. The Tc-99m ECD SPECT demonstrated only small foci of hypoperfusion at bilateral hemispheres with the coefficient of variation as 10.8%. (B) A 39-year-old female had initial presentations of depressive mood and seizure. She had positive ACA IgG, APhL IgG, anti-β2GPI IgG and lupus anticoagulants. There was no definite organic brain change in the brain MRI; however, the Tc-99m ECD SPECT revealed large areas of hypoperfusion at bilateral hemispheres with the coefficient of variation up to 20.9%. Ten months later, she developed an ischemic stroke.

Each participant received an intravenous injection of 740 to 1110 MBq 99mTc-ethyl cysteinate dimer (Neurolite, Institute of Nuclear Energy Research, Taiwan), which has more rapid blood clearance and provides better target-to-background ratio compared to formerly-used agent, Tc-99m hexamethylpropyleneamine oxime (HMPAO) [12]. Twenty minutes later, image acquisition was started with patients lying in a dark and quiet room on a GE Infinia Hawkeye (GE Healthcare, Waukesha, WI, USA) or Siemens Symbia T2 (Siemens Healthcare Systems, Erlangen, Germany) dual-head gamma camera. Fan-beam collimators were used for the GE Infinia Hawkeye, with an energy window setting of 140 keV (±7%). Imaging data were obtained with the 360° acquisition for 60 projections at 20 seconds/projection in a 128×128 matrix and processed in an XELERIS Workstation (Version 1.1362) using filtered back projection (FBP) reconstruction with a Butterworth filter (order 10, cut-off 0.45). Low energy and high resolution parallel hole collimators were used for the Siemens Symbia T2, with an energy window setting of 140 keV (±10%). After 360° image acquisition with 64 projections at 30 seconds/projection in a 128×128 matrix, the data were processed in a Syngo Workstation (Version 7.0.7.14), using FBP reconstruction with a Metz filter. Attenuation correction was performed by the method of Chang (GE Infinia Hawkeye) or CT attenuation map (Siemens Symbia T2) [13]. Each study was reoriented with the Talairach space provided by the NeuroGam Software package (GE Medical Systems, Segami Corporation, Columbia, MD, USA). Regions of interest were classified by the brain lobes. The activity of each voxel in the specified region was then quantified and compared with the maximal uptake of the cerebellum; the results were expressed as percentages. Heterogeneity of brain perfusion was measured as the coefficient of variation, which was defined as the ratio of the standard deviation to the mean. The illustrations of the brain perfusion scan were presented in Fig 1.

### Statistical analysis

The data collected during this study are presented in summary tables and figures. Continuous variables were summarized by mean, SD, median, minimum and maximum and categorical variables were summarized by percentages. Analysis of variance (ANOVA) was applied to evaluate the difference of brain perfusion between groups, and a *P* value of <0.05 was considered statistically significant. All statistical analyses were performed using Stata 14.2 (Stata Crop, College Station, Texas, USA).

## Results

**Fig 2:**
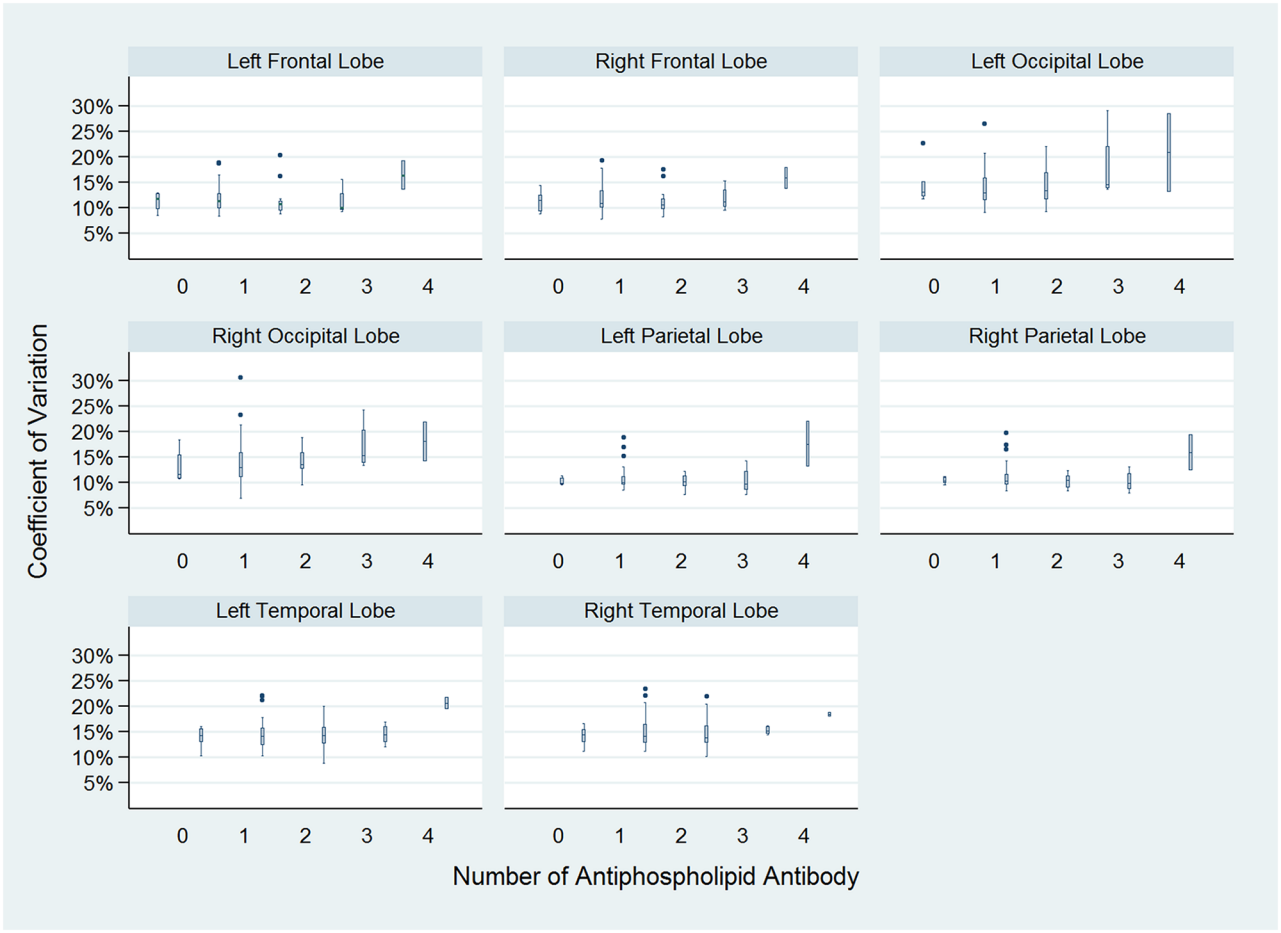
Heterogeneity of brain perfusion. The coefficient of variation of Tc-99m ECD SPECT signals is positively associated with the number of antiphospholipid antibodies (*P* = 0.01). The boxes represent interquartile range (IQR). The horizontal lines in the boxes represent median values; the horizontal lines out of the boxes represent 1.5 IQR. The black dots are outliers.

Total 60 adult patients were included in this study; fifty-two (86.7%) patients were women, and 8 (13.3%) patients were men. The mean age was 38.3 ± 11.5 years. In the case group, 31 (51.7%) patients carried one antiphospholipid antibody, 17(28.3%) carried two antiphospholipid antibodies, 4(6.7%) carried three antiphospholipid antibodies, and 2 (3.3%) carried four antiphospholipid antibodies. Six (10.5%) patients without any positive antiphospholipid antibody served as the control group. The baseline characteristics of the subjects are shown in Table 1. The most common neuropsychiatric manifestation was headache (n = 34; 56.7%) and the second was dizziness (n = 25; 41.7%); the clinical neuropsychiatric manifestations of the patients are shown in Table 2. The traditional brain image studies including CT (n = 25; 41.7%) and MRI (n = 30; 50.0%) did not reveal definite organic brain change in these patients. In brain SPECT studies, there was no significant difference of mean brain perfusion between groups (*P* = 0.69); however, the Tc-99m ECD SPECT revealed significant heterogeneity of brain perfusion in relation to the number of antiphospholipid antibodies (*P* = 0.01). The coefficient of variation of brain perfusion, by the groups, is shown in Fig 2.

**Table 1:** Baseline characteristics of patients.

	APA = 0 (n = 6)	APA = 1 (n = 31)	APA = 2 (n = 17)	APA = 3 (n = 4)	APA = 4 (n = 2)	Total (n = 60)
Age (years)	32.5 ± 11.2	38.6 ± 12.2	39.3 ± 11.3	41.0 ± 11.0	36.8 ± 3.4	38.3 ± 11.5
Female	6 (100.0)	28 (90.3)	14 (82.4)	2 (50.0)	2 (100.0)	52 (86.7)
BMI (kg/m^2^)	21.0 ± 3.8	23.3 ± 5.2	23.4 ± 5.7	21.5 ± 0.7	26.1 ± 7.8	23.0 ± 5.1
Hypertension	0 (0.0)	2 (6.5)	2 (11.8)	0 (0.0)	0 (0.0)	4 (6.7)
Diabetes Mellitus	0 (0.0)	1 (3.2)	1 (5.9)	0 (0.0)	1 (50.0)	3 (5.0)
Hyperlipidemia	0 (0.0)	1 (3.2)	2 (11.8)	0 (0.0)	0 (0.0)	3 (5.0)
Ever-smoker	1 (16.7)	6 (19.4)	1 (5.9)	0 (0.0)	0 (0.0)	8 (13.3)

Values are n (%) or mean ± SD.

APA: antiphospholipid antibody; BMI: body mass index.

**Table 2:** Neuropsychiatric manifestations of patients.

	APA = 0 (n = 6)	APA = 1 (n = 31)	APA = 2 (n = 17)	APA = 3 (n = 4)	APA = 4 (n = 2)	Total (n = 60)
Headache	5 (83.3)	17 (54.8)	8 (47.1)	3 (75.0)	1 (50.0)	34 (56.7)
Dizziness	5 (83.3)	12 (38.7)	6 (35.3)	2 (50.0)	0 (0.0)	25 (41.7)
Vertigo	1 (16.7)	2 (6.5)	2 (11.8)	0 (0.0)	0 (0.0)	5 (8.3)
Seizure	1 (16.7)	1 (3.2)	1 (5.9)	0 (0.0)	1 (50.0)	4 (6.7)
Depression	0 (0.0)	12 (38.7)	4 (23.5)	0 (0.0)	1 (50.0)	17 (28.3)
Psychosis	2 (33.3)	4 (12.9)	3 (17.6)	0 (0.0)	0 (0.0)	9 (15.0)

Values are n (%).

APA: antiphospholipid antibody.

## Discussion

APS spectrum includes thromboembolism, pregnancy morbidity and non-criteria manifestations; most previous studies of APS focused on thrombosis or complications during pregnancy [4]. Nevertheless, non-criteria presentations can also severely affect the patients’ health. Due to only a limited number of clinical trials for non-criteria APS, there is no consensus regarding how to manage non-criteria presentations of APS [14, 15]. The non-criteria neuropsychiatric presentations of APS include headache, seizure, psychosis, etc [5–8]. In addition, one study found that 40% of antiphospholipid antibody carriers without major brain MRI abnormalities had cognitive impairment, which was determined by the global cognitive impairment index [16]. However, there were still no objective methods qualified to detect the early central nervous system involvement in APS. Thus, we evaluated the usefulness of Tc-99m ECD SPECT for this purpose.

Antiphospholipid antibodies are the risk factors for ischemic stroke and myocardial infarction, and patients with antiphospholipid antibodies are in a prothrombotic state [17–20]. Several mechanisms are involved in the development of the APS prothrombotic state; initially, antiphospholipid antibodies interact with the plasma membrane binding protein, β2GPI, on platelets and endothelial cells, and after that the intracellular signal cascades are initiated. Nuclear factor кB and p38 mitogen-activated protein kinase play key roles in the cascade, and they results increased production and expression of adhesion molecules (intercellular adhesion molecule 1, vascular cell adhesion molecule 1 and E-selectin), tissue factors, chemokines (monocyte chemotactic protein 1), and inflammatory cytokines (interleukin 4, interleukin 6, interleukin 8 and tumor necrosis factor alpha) [21–23]. Furthermore, the binding between antiphospholipid antibodies and β2GPI will initiate the process of the classical complement cascade; complement component 5a will be generated and then activate neutrophils [24]. Previous studies showed that the risk of thromboembolic events is proportional to the number of antiphospholipid antibodies which the patient carries, and the rate was 5.30% per year for triple-positive carriers compared to 1.36% for single-positive carriers [25]. Therefore, we classified the subjects according to the number of antiphospholipid antibodies they carried.

Previous studies demonstrated that SPECT was able to detect the decreased brain blood flow in APS patients who had normal brain MRI findings, and SPECT could be used to monitor the treatment response in patients who received anticoagulants [11]. Despite that, using SPECT to detect the early central nervous system involvement in non-criteria APS patients has not yet been evaluated. In our study, the mean brain perfusion did not significantly differ between the groups, which indicates that the conventional parameters lack enough sensitivity to detect early abnormalities in microcirculation. In addition to various image modalities, image biomarkers of heterogeneity are increasingly used for several disease entities. In nuclear medicine researches, related parameters were used to investigate tumor type, grade, prognosis and therapeutic response in cancer patients [26–28]. Except that, heterogeneity was also applied to evaluate brain metabolism in neurodegenerative diseases, such as Alzheimer's disease [29]; among the different measurement methods, the coefficient of variation is a relatively clear and readily available biomarker in current software packages [30–32]. In our study, Tc-99m ECD SPECT demonstrated a significant positive association between the heterogeneity of brain perfusion and the number of antiphospholipid antibodies the patients carried.

There are some limitations should be evaluated in this hospital-based retrospective study. First, the control group was not from a healthy population. The strength of the association between antiphospholipid antibodies and heterogeneity of brain perfusion may be underestimated due to this potential selection bias. Second, the neuropsychiatric presentations of the subjects were not identical; therefore, the cases selected in this study could not well represent a homogeneous group of non-criteria APS. Third, systemic lupus erythematosus (SLE) was frequently accompanied with APS [33], and SLE patients with CNS involvement could have abnormalities in brain images. Hence, it is a concern that these patients with non-criteria APS might be complicated with SLE. In order to control this confounding factor, the patients with any positive antibody to extractable nuclear antigens were excluded carefully in this study.

One of the difficulties would be met when conducting a non-criteria APS clinical trial is which outcome end points should be used. If a thromboembolic event is the primary end point of non-criteria APS clinical trials, it could be expected that a huge sample size and long study duration would be needed to detect the potential treatment effect. If the heterogeneity of brain perfusion is a reliable surrogate endpoint, it will be easier to evaluate the outcomes in the studies. To our knowledge, this is the first study demonstrating the usefulness of assessing heterogeneity of brain perfusion in antiphospholipid antibody carriers. We are looking forward to prospective studies evaluating the effectiveness of heterogeneity of brain perfusion as a marker to predict thromboembolic events.

## Conclusions

In summary, our findings suggest that Tc-99m ECD SPECT can detect the increased heterogeneity of brain perfusion in the non-criteria antiphospholipid antibody carriers with neuropsychiatric manifestations; furthermore, the heterogeneity of brain perfusion is in relation to the number of antiphospholipid antibodies patients carry.

## Supporting information

S1 Data The raw data of Tc-99m ECD SPECT in this study. (XLSX)

S1 Table The distribution of antiphospholipid antibodies. (DOCX)
