# Supplementary material for: Increased heterogeneity of brain perfusion is an early marker of central nervous system involvement in antiphospholipid antibody carriers
Source: PLoS One. 2017 Aug 1;12(8):e0182344. doi: 10.1371/journal.pone.0182344 (PMC5538638; doi:10.1371/journal.pone.0182344)
Supplement: S1 Table — (DOCX) [file pone.0182344.s002.docx]

**S1 Table. The distribution of antiphospholipid antibodies.**

|  | **APA**=**1** | **APA**=**2** | **APA**=**3** | **APA**=**4** |
| --- | --- | --- | --- | --- |
|  | **(n=31)** | **(n=17)** | **(n=4)** | **(n=2)** |
| ACA IgM | 10 (32.3) | 14 (82.4) | 4 (100.0) | 0 (0.0) |
| ACA IgG | 4 (12.9) | 5 (29.4) | 2 (50.0) | 2 (100.0) |
| Anti-β2GPI IgG | 8 (25.8) | 0 (0.0) | 2 (50.0) | 2 (100.0) |
| APhL IgM | 7 (22.6) | 13 (76.5) | 3 (75.0) | 0 (0.0) |
| APhL IgG | 2 (6.5) | 2 (11.8) | 0 (0.0) | 2 (100.0) |
| Lupus anticoagulant | 0 (0.0) | 0 (0.0) | 1 (25.0) | 2 (100.0) |

Values are n (%).

APA: antiphospholipid antibody.
